# Characterization of Two Novel Variants of the Steroidogenic Acute Regulatory Protein Identified in a Girl with Classic Lipoid Congenital Adrenal Hyperplasia

**DOI:** 10.3390/ijms21176185

**Published:** 2020-08-27

**Authors:** Efstathios Katharopoulos, Natascia Di Iorgi, Paula Fernandez-Alvarez, Amit V. Pandey, Michael Groessl, Shraddha Dubey, Núria Camats, Flavia Napoli, Giuseppa Patti, Marilea Lezzi, Mohamad Maghnie, Christa E. Flück

**Affiliations:** 1 Department of Paediatrics, Division of Endocrinology, Diabetology & Metabolism, Bern University Hospital, 3010 Bern, Switzerland; Efstathios.Katharopoulos@dbmr.unibe.ch (E.K.); amit@pandeylab.org (A.V.P.); shriddha4@gmail.com (S.D.); 2 Department of Biomedical Research, Bern University Hospital and University of Bern, 3010 Bern, Switzerland; michael.groessl@insel.ch; 3 Graduate School Bern, University of Bern, 3012 Bern, Switzerland; 4 Department of Paediatrics, Istituto Giannina Gaslini, University of Genoa, 16147 Genoa, Italy; natasciadiiorgi@gaslini.org (N.D.I.); flavianapoli@gaslini.org (F.N.); giusypattis@gmail.com (G.P.); lezzimarilea@gmail.com (M.L.); MohamadMaghnie@gaslini.org (M.M.); 5 Department of Neuroscience, Rehabilitation, Ophthalmology, Genetics, Maternal and Child Health, University of Genova, 16147 Genoa, Italy; 6 Department of Clinical and Molecular Genetics and Rare Disease Unit, University Hospital Vall d’Hebron, 08035 Barcelona, Spain; paufernandez@vhebron.net; 7 Department of Nephrology and Hypertension, Bern University Hospital, 3010 Bern, Switzerland; 8 Growth and Development Research Unit, Vall d’Hebron Research Institute (VHIR), Centre of Biomedical Research on Rare Diseases (CIBERER), Instituto de Salud Carlos III, 08035 Barcelona, Spain; nuria.camats@vhir.org

**Keywords:** adrenal insufficiency, StAR, steroidogenic acute regulatory protein, lipoid congenital adrenal hyperplasia, rare disease

## Abstract

Congenital adrenal hyperplasia (CAH) consists of several autosomal recessive disorders that inhibit steroid biosynthesis. We describe a case report diagnosed with adrenal insufficiency due to low adrenal steroids and adrenocorticotropic hormone excess due to lack of cortisol negative feedback signaling to the pituary gland. Genetic work up revealed two missense variants, p.Thr204Arg and p.Leu260Arg in the *STAR* gene, inherited by both parents (non-consanguineous). The StAR protein supports CYP11A1 enzyme to cleave the side chain of cholesterol and synthesize pregnenolone which is metabolized to all steroid hormones. We used bioinformatics to predict the impact of the variants on StAR activity and then we performed functional tests to characterize the two novel variants. In a cell system we tested the ability of variants to support cholesterol conversion to pregnenolone and measured their mRNA and protein expression. For both variants, we observed loss of StAR function, reduced protein expression and categorized them as pathogenic variants according to guidelines of the American College of Medical Genetics and Genomics and the Association for Molecular Pathology. These results fit the phenotype of the girl during diagnosis. This study characterizes two novel variants and expands the list of missense variants that cause CAH.

## 1. Introduction

Congenital adrenal hyperplasia (CAH) was first described by De Crecchio in 1865 [1], and is defined by the inability to produce sufficient amounts of glucocorticoids by the adrenal glands. CAH is the umbrella term for several autosomal recessive congenital disorders of the adrenal cortex that manifest with impaired adrenal steroidogenesis due to pathogenic variants in genes encoding steroidogenic enzymes or cofactors [2]. In Europe, CAH is mostly caused by variants in the *CYP21A2* gene, which results in impaired 21-hydroxylase activity. Rarer forms of CAH are due to pathogenic variants in all other genes involved in the biosynthesis of glucocorticoids, such as *STAR*, *CYP11A1*, *HSD3B2*, *CYPB11B1*, *CYP17A1* and *POR* [2].

Lipoid CAH (OMIM: 201710) is a form of CAH that was described by Prader and Gurtner in 1955 [3] in a girl with 46,XY sex reversal and fatal adrenal insufficiency (AI). Later studies revealed that it is caused by pathogenic variants in the gene for steroidogenic acute regulatory protein (*STAR*, OMIM: 600617) [4,5]. StAR facilitates the transfer of cholesterol from the outer (OMM) to the inner mitochondrial membrane (IMM), and accounts for about 86% of cholesterol import [6]. Two protein complexes have been discovered to aid cholesterol transfer to the IMM: a smaller 66 kDa complex including MDH2, IDH2, P450scc and ADXR, and a larger 800 kDa complex consisting of at least eight proteins, including VDAC, ACAT1 and ATAD3A [7]. Only about 14% of cholesterol import occurs in a StAR-independent manner [8]. In the inner mitochondrial membrane, cholesterol is converted to pregnenolone by the synergistic action of CYP11A1 enzyme with adrenodoxin reductase and adrenodoxin cofactors. This first and rate-limiting step is very important as pregnenolone is the essential precursor for the biosynthesis of all steroids [9,10]. Thus, inactivating variants of StAR impede the production of pregnenolone and therefore all steroidogenesis. StAR deficiency affects steroid production in gonads and adrenal glands, but not in the placenta, which does not express *STAR* [11]. Mitochondrial cholesterol trafficking in placenta cells seems to depend on MLN64 (STARD3). MLN64 is an endosomal protein, which lacks StAR activity, but binds cholesterol [12]. Presumably, MLN64 is processed in vivo with the removal of 218 amino acids at the N-terminus, resulting in a 227 amino acids (in the C-terminus) protein called N-218 MLN64. This cleaved product has a StAR-like activity, as it shares 37% sequence identity and 50% sequence similarity with StAR [13,14,15].

Like other forms of CAH, lipoid CAH can be subdivided in classic and non-classic forms. The severe classic lipoid CAH is life-threatening soon after birth due to (almost) complete loss of StAR activity causing severe glucocorticoid and mineralocorticoid deficiency. Its pathomechanism is best described by the two-hit model proposed by Miller [16]. The first hit consists of the actual loss of StAR activity, which leads to accumulation of cholesterol and cholesterol esters in steroidogenic cells and to the enlargement of the adrenal glands [17]. The second hit occurs later in life with the lipid overload. Accumulated lipid droplets destroy directly or indirectly (through sterol auto-oxidation products) steroidogenic cells and result in loss of the remaining StAR-independent cholesterol transport (~14%) [5,16].

Non-classic lipoid CAH patients with non-deleterious genetic variants retain 10%–25% of StAR activity and have therefore a milder phenotype with later on-set of the disease. Patients often show no symptoms until puberty [18,19,20]. These cases are initially often (mis-)diagnosed with familial glucocorticoid deficiency (FGD), which is actually caused by several genetic disorders that lead to AI and adrenocorticotropic hormone (ACTH) resistance only, without affecting gonadal steroidogenesis and thus sex development [2,19]. By contrast, patients with severe lipoid CAH present with AI and salt wasting crisis soon after birth. In addition, karyotypic 46,XY males present with normal female looking external genitalia. Lack of aldosterone increases renal salt wasting and renin plasma levels [5,16,20]. Absence of cortisol leads via negative feedback to the hypothalamus and the pituitary gland to excessive ACTH secretion. As ACTH is a growth factor for the adrenal cortex, this will lead to adrenal hyperplasia mediated through its receptor MC2R. Moreover, excess ACTH stimulates the MC1R and results in skin hyperpigmentation. StAR deficiency also affects sex steroid production. In severely affected 46,XY individuals, loss of StAR activity leads to lipid accumulation in Leydig cells. Lipid accumulation results in cell destruction and lack of testosterone biosynthesis, which is essential for male sex development early in fetal life. Therefore, these individuals will manifest at birth with ambiguous or female external genitalia [16]. By contrast, 46,XX individuals (even with a severe form of lipoid CAH) do not encounter problems related to sex steroid production until puberty or later because female sex steroid production is minimal during fetal life and prepubertally. Thus, the second hit, which destroys the gonad in karyotypic boys already *in utero*, will hit the girls much later. However, most 46,XY and 46,XX individuals with severer forms of StAR deficiency will have difficulties with pubertal development, while with milder forms gonadal function may be normal for longer, especially in 46,XX women [5,21,22]. Therapy of lipoid CAH consists of replacement of steroid hormones as soon as the diagnosis is made and may include glucocorticoids, mineralocorticoids and sex hormones as defined for each individual patient and his actual situation [20].

So far, more than 190 patients with *STAR* variants have been described. Almost 100 patients are reported in the Japanese literature as StAR deficiency is the most common form of CAH in Japan. Population genetics showed an association of the *STAR* variant p.Q258X to the Japanese and Korean population and p.R182L to Palestinian Arabs [23]. In this study, we describe a 46,XX patient who manifested with clinical and biochemical signs of early AI. The patient was investigated for disease-causing genetic variants [2]. Two novel variants in the *STAR* gene were found and characterized in great detail by bioinformatics predictions and functional in vitro studies.

## 2. Results

### 2.1. Genetic Identification of Novel Variants in StAR

**Figure 1:**
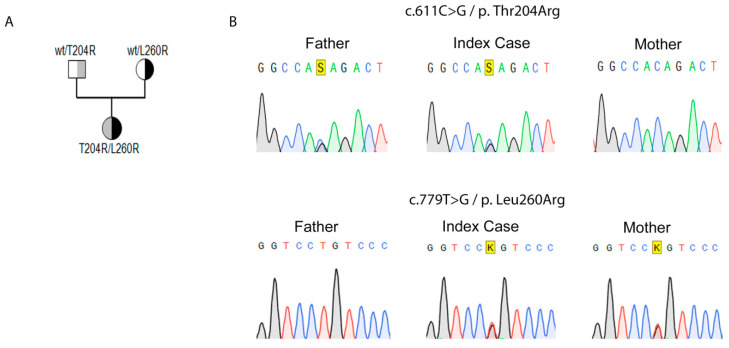
Genetic work up of the family. (**A**) Family pedigree. The c.611C>G, p.Thr204Arg was paternally inherited and the c.779T>G, p.Leu260Arg was maternally inherited. The patient is compound heterozygous for both mutations. (**B**) Electrograms of the variants identified in index patient and her parents with heterozygote base pairs highlighted.

Genomic DNA isolated from the patient was subjected to genetic analysis using a defined custom gene panel for AI on a Next Generation Sequencing (NGS) platform [24]. Two novel missense variants c.611C>G, (p.Thr204Arg) and c.779T>G, (p.Leu260Arg) were detected in the *STAR* gene and confirmed by direct Sanger sequencing. Family analysis revealed that the c.611C>G, (p.Thr204Arg) variant was paternally-inherited and the c.779T>G, (p.Leu260Arg) variant was maternally-inherited (Figure 1). These variants have not been reported in the literature previously, nor were they found in population data repositories (GnomAD).

### 2.2. STAR Conservation Analysis and Mutagenesis Prediction for T204R and L260R

**Figure 2:**
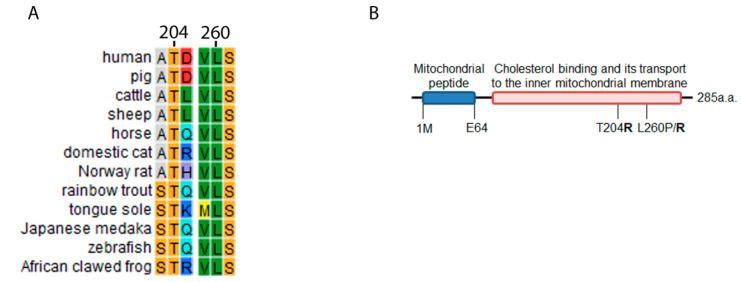
(**A**) Conservation analysis showing that amino acid positions T204 and L260 are highly conserved among species and important for StAR function. (**B**) The StAR protein has 285 amino acids. The variants studied in this work lie within the functional area of the protein, towards the C-terminus. The N-terminus contains the lead sequence, which directs StAR to mitochondria.

We performed a conservation analysis for the *STAR* gene and computational mutagenesis prediction for variants p.T204R, p.L260R and p.L260P to predict the effect on protein function. To find how conserved the *STAR* gene is, we built a custom position specific scoring matrix (PSSM) using four rounds of iterated PHI-BLAST search. This showed that both amino acid positions p.T204 and p.L260 are conserved among species (Figure 2). We therefore expected that any variants at these positions would be disease causing. To evaluate their pathogenicity *in silico*, we analyzed the variants in the SDM predictor mutagenesis software. Variants p.T204R, p.L260R and p.L260P revealed lower values of −1.15, −2.75 and −4.31 indicating a higher chance of pathogenicity. Considering that p.T204 is located away from the cholesterol binding site in the StAR structure, we expected variants at this position to be less damaging as compared to variants at position p.L260, which is located near the cholesterol binding site.

### 2.3. Use of StAR Structural Model, Computational Mutagenesis and Simulation of Interaction with Cholesterol for Variant Prediction

**Figure 3:**
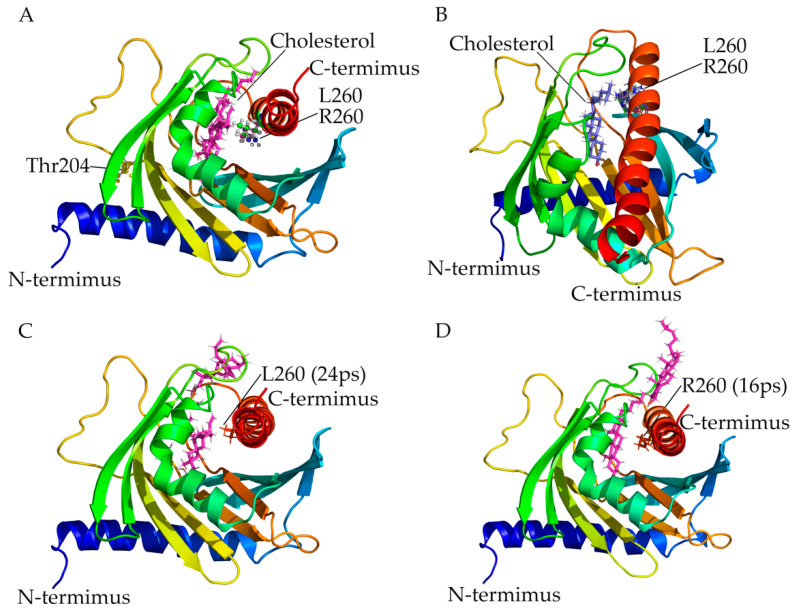
Simulated StAR variants on the StAR 3D model. The N-terminus is depicted with a blue color and the C-terminus with red color. The cholesterol molecule is shown in magenta (**A**) Threonine 204 is shown and both leucine (in green) and arginine (in blue) are depicted in the structure. (**B**) The Arg260 side chain was flexible during simulation and could be seen in different conformations. (**C**,**D**) The p.L260R variant in StAR (**C**) seem to impact the binding of cholesterol as evidenced by lower time required to pull the cholesterol out of the wt StAR (**D**).

We created structural models of amino acid changes caused by mutations in StAR using the X-ray crystal structure available in PDB database (3PLO). Amino acid analysis revealed that leucine 260 is located near the cholesterol binding site and its mutation may impact the binding and release of cholesterol (Figure 3A). We have previously shown that a mutation of leucine 260 to proline had an adverse impact on the StAR function. When leucine 260 was mutated to arginine, a larger and polar residue, an impact on protein stability as well as binding of cholesterol was predicted. During the course of MD simulation, arginine 260 side chain was flexible and could be seen in multiple conformations (Figure 3B), indicating an impact on potential entry and binding of cholesterol as well as protein stability. One or both of these factors may contribute towards the lower functional activity of p.L260R variant of the StAR protein.

To understand the impact of p.L260R variant on the entry and exit of cholesterol, we performed a steered molecular dynamic simulation, by applying a constant pulling force to the cholesterol molecule during simulation. The p.L260R variant in StAR seems to impact the binding of cholesterol as evidenced by lower time required to pull the cholesterol out of the StAR (16 ps, compared to 24 ps needed for the wild-type (wt) StAR protein) (Figure 3C,D).

For the p.T204R variant of StAR, a direct impact on cholesterol binding and ability of StAR to transport cholesterol can be ruled out. Based on structural analysis, impact on protein stability and expression levels of the variant protein, it likely leads to lower cholesterol transport into the mitochondria. In addition, the amino acid threonine 204 is located into a recognition site for multiple kinases (AGMATDFGN), and phospho site prediction analysis shows recognition for Cam-II, GSK3, cdc2 and p38MAPK. It is likely that loss of protein phosphorylation at threonine 204 impacts the in-situ protein stability or function of the StAR protein.

### 2.4. Functional Testing of Novel StAR Variants In Vitro

**Figure 4:**
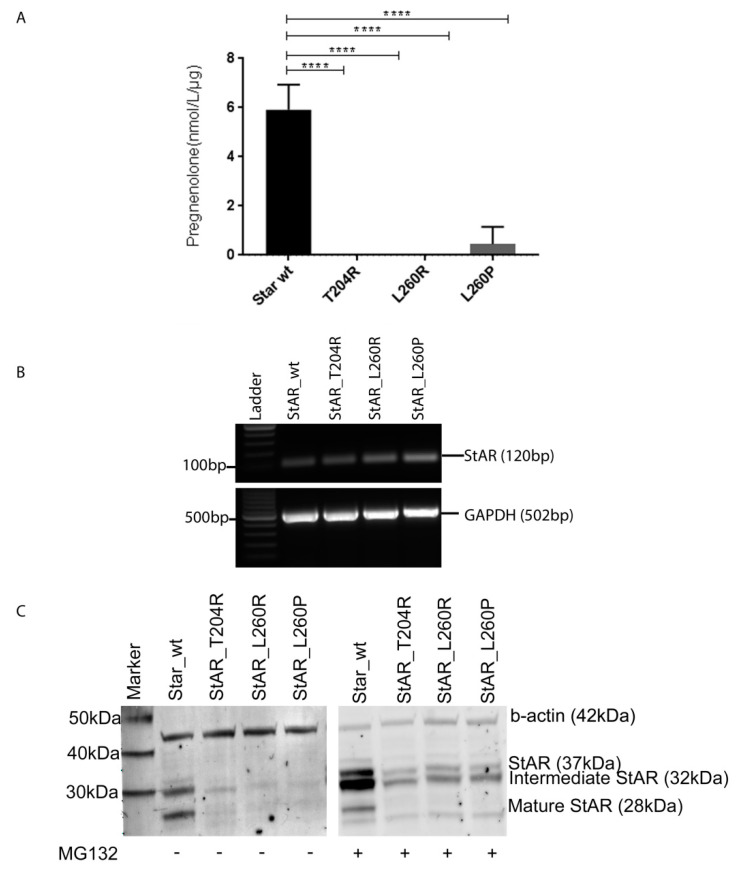
We evaluated the activity of wt StAR and variants to support conversion of cholesterol to pregnenolone in a COS1 cell model. (**A**) To determine StAR activity, we measured pregnenolone production 48 h after transfection of COS1 cells with StAR vectors and vectors to express the side-chain-cleavage system (CYP11A1/ADX/ADXR). StAR activity of wt and variants is shown as fold change compared to wt and normalized to empty vector and total protein. **** *p* < 0.0001 (**B**) Expression level of wt StAR and variants in transfected COS1 cells (**C**) Protein expression of wt and variants StAR in COS1 cells treated with (+) and without (−) proteasome inhibitor MG132 assessed by Western blot.

To determine the functional consequences of the two novel variants of StAR protein, we transfected COS1 cells with wt StAR or variants and the CYP11A1/ADXR/ADX steroidogenic machinery, and assessed pregnenolone production with LC-MS/MS. We found that both StAR-T204R and StAR-L260R variants lost activity to support steroidogenesis in this assay completely (Figure 4A). For comparison, we also tested the previously characterized variant StAR-L260P, which also only showed minimal StAR activity (~8%) (Table 1) [25].

The mRNA expression of the variants, assessed by RT-PCR, was similar to that of wt StAR (Figure 4B). In Western blot analysis, all StAR variants (StAR-T204R, StAR-L260R and StAR-T260P) showed lower amount of protein expression compared to wt (Figure 4C). We therefore treated transfected COS1 cells with proteasome inhibitor MG132 to assess whether protein degradation was different in variants. MG132 treatment increased the total protein amount of wt StAR and all StAR variants; however, wt increment was relatively bigger (Figure 4C). In addition, MG132 treatment affected the three isoforms of StAR (immature—37 kDa, intermediate—32 kDa and mature—28 kDa) differently having biggest effect on the 32 kDa isoform (Figure 4C). Thus, proteasome degradation may only partially explain the observed difference in protein expression of StAR variants.

## 3. Discussion and Conclusions

Lipoid CAH is a very rare congenital disease that without timely diagnosis and treatment may be lethal. In this work, we describe a newborn 46,XX girl who was diagnosed with classic lipoid CAH after manifesting with primary AI soon after birth. Her clinical manifestation was typical for primary AI and genetic analysis by NGS revealed novel variants in the *STAR* gene inherited by both parents. Bioinformatics and functional studies confirmed the disease-causing effect as suggested by current recommendations of ACMG-AMP guidelines [26]. In vitro testing of identified variants p.T204R, p.L260R and p.L260P revealed significant loss of enzymatic activity. Protein expression level for these variants was significantly reduced compared to wt StAR, but mRNA expression level was similar. Previous studies have shown lower protein expression of StAR variants p.N148K, p.A218V, p.E169K and p.L275P [16,27,28]. Specifically variant p.N148K was shown to fold in a different way, which changed its binding affinity to mitochondria [28]. Our bioinformatics studies showed that identified StAR variants have lower protein stability compared to wt protein. We therefore considered proteolytic degradation as possible cause for the difference between mRNA and protein expression. To address this question, we treated cells with proteasome inhibitor MG132 and found minor differences in the expression pattern of wt StAR and variants. However, the observed difference may not fully explain the lower protein levels due to the fact that protein degradation also involves the lysosomal pathway, which has not been tested [29].

Although severe StAR deficiency affects adrenal and gonadal steroidogenesis, 46,XX newborns only show AI early and do not show problems in sex development until puberty, when ovarian steroidogenesis is supposed to be activated. At puberty, some girls with *STAR* variants even manage to proceed through pubertal development and have regular menstrual cycles [30,31], but early cessation resulting in ovarian cysts and infertility is then seen [16,31]. We suggest that using current fertility techniques, such as egg freezing, early could provide a chance for these girls to preserve fertility [32,33]. Pregnancy has been successfully achieved in other disorders of steroidogenesis affecting fertility such as CYP17 deficiency [34]. The situation of fertility is completely different in severely affected 46,XY babies, in which gonadal steroidogenesis is affected during fetal life and development of male typical external genitalia is absent (see also Introduction) [22]. In fact, these 46,XY babies with typical female external genitalia are mostly assigned to female sex at birth and live in the female gender [3,25]. At puberty, sex hormone replacement therapy is then necessary.

In the ClinVar database there are 60 annotated single nucleotide polymorphisms in the *STAR* gene responsible for classic and non-classic lipoid CAH. This includes 23 exonic variants, of which 15 have been categorized as pathogenic/likely pathogenic. Our patient was compound heterozygous for two novel variants causing severe StAR deficiency. The parents, who were not related, were heterozygous for one of the variants with no clinical signs. Variants p.T204R and p.L260R showed complete loss of StAR activity in our functional assays, thus placing our patient in the group of classic lipoid CAH. Other variants, such as p.R188C and p.V187M that retain 17% and 28% of StAR activity respectively, were identified in patients primarily manifesting with AI later in life and are therefore classified as non-classic lipoid CAH [18]. In these cases, even 46,XY individuals are born as typical males with normal male external genitalia due to the remaining StAR activity.

Despite the fact that several *STAR* variants have been described in lipoid CAH literature, the clinical picture of the patients is quite variable in classic and non-classic lipoid CAH. Moreover, our current understanding of the mechanism of mitochondrial cholesterol transport is limited to StAR function, despite several other proteins other than StAR seem to participate [8]. It is therefore possible that the observed heterogeneity may be influenced by modulating factors on transport capacity of cholesterol and hence steroidogenesis.

Proteins that mediate mitochondrial cholesterol transport in a vesicular or non-vesicular manner are the following: (1) STAR-related lipid transfer (START) domain proteins (for non-vesicular transfer), and (2) soluble NSF attachment protein receptor (SNARE) protein complexes (for vesicular transfer). Five out of 15 START domain proteins bind cholesterol. StAR (or STARD1) and MLN64 (or STARD3) have been described before (see Introduction). STARD4 and its two homologues STARD5 and STARD6 transport intracellular sterol and also bind cholesterol. In fact, STARD6 had similar or greater activity than StAR in vitro [8]. Stable reduction of STARD4 in human osteosarcoma epithelial cells led to cellular cholesterol increase and decreased sterol transport. Reduction of STARD4 also decreased plasma membrane order and lowered the generalized polarization value compared to control, as was quantified with fluorescent microscopy. Overexpression of STARD4 rescued the plasma membrane defect [35]. STARD5 has been shown to mediate cholesterol transport between liposomes and between plasma membrane and endoplasmic reticulum (ER). In the same study deletion of STARD5 in mice resulted in reduced plasma membrane cholesterol, lipid accumulation but unaltered cholesterol levels intracellularly in macrophages (which express STARD5) [36]. In addition, STARD5 seems also important for bile acid transportation [8]. Overall, START domain proteins play a crucial role in sterol and cholesterol homeostasis. They are predominantly expressed in liver and kidney, as well as steroidogenic tissues. However, their expression in other tissues challenges their role in mitochondrial cholesterol transport and steroid biosynthesis [8,37]. Unlike START proteins, SNARE protein complexes do not bind cholesterol, but facilitate its vesicular transport by bridging lipid droplets to OMM. This particular cholesterol transport mechanism may compensate for StAR deficiency in patients with classic lipoid CAH who accumulate lipid droplets in steroidogenic cells. Adrenocortical cells carry a larger lipid load than gonadal Leydig cells, and thus, SNARE contribution might differ among steroidogenic tissues in pathologic conditions [8]. However, the exact role of START and SNARE proteins in steroid biosynthesis remains unknown and their possible modulating effect on the phenotype of lipoid CAH speculative.

In summary, our work expands the list of *STAR* variants that cause classic lipoid CAH. The reported girl presented very early with typical symptoms of AI and was diagnosed and treated timely due to readily available genetic NGS testing. Although StAR deficiency may affect adrenal and gonadal steroidogenesis severely, current treatment options allow a normal life and may even enable fertility with the aid of newer preservation techniques.

## 4. Materials and Methods

### 4.1. Case Report

The girl was born at term to non-consanguineous parents (mother from Ecuador, father from South Italy) by Caesarean section for maternal hypertension. Birth weight was 2980 g (−0.21 SDS) [38], birth length 48 cm (−0.39 SDS) and head circumference 33 cm (−0.47 SDS). No problems were reported during neonatal period. Physical exam was normal and external genitalia were typical female.

At 45 days of life, she presented with fever, vomiting and diarrhea. Treatment with amoxicillin and betamethasone was started without clear diagnosis by a primary care physician, and symptoms improved. However, after stopping oral steroids, vomiting reoccurred and she was noted to have rather low serum sodium and high potassium under fluid replacement therapy (Table 2). Additional lab exams showed massively elevated ACTH, increased renin activity and aldosterone in the lower range, and all measured adrenal steroids were mostly undetectable (Table 2). Thus, adrenal insufficiency was diagnosed and replacement therapy with hydrocortisone (~40 mg/m^2^/day) and fludrocortisone (0.1 mg/day) was started.

The patient was then referred to a third-level center for further diagnostic workup. Physical examination was mostly unremarkable, but revealed pigmentation of the skin. Typical female external genitalia were noted without signs of virilization. Blood pressure and heart rate were within normal range. Ultrasound showed normal sized adrenals. Additional oral sodium chloride therapy was started (30 mEq/day) and then modulated according to clinical and laboratory parameters in the course. Further diagnostic exams revealed a normal 46,XX female karyotype (with absence of SRY region by FISH analysis of 100 nuclei at interphase). Very long chain fatty acids were normal. Anti-adrenal antibodies were negative. Diagnosis was confirmed by plasma steroid profile at six months; blood sample was taken 12 h after last dose of oral hydrocortisone and 24 h after last dose of oral fludrocortisone.

During follow-up, steroid replacement doses were adjusted to growth and lab exams. Growth was normal. She is now 3 years old and height is +1.1 SDS (+2.1 SDS greater than target height), height velocity 10.1 cm/years (SDS +1.3) [39]. BMI is in the obesity range for age (+2.1 SDS) [40]. Bone age corresponds to chronological age. Mild hypertension was noted, but normalized after stopping sodium chloride supplementation, and echocardiography was normal. In addition, the girl showed idiopathic premature thelarche with normal findings of ovaries and uterus by ultrasound, but slightly elevated FSH (5.2 U/L, ref. values 0.5–3.7), but not measurable LH and 17β-estradiol consistent with idiopathic premature thelarche. Furthermore, the girl is monitored for mild psychomotor delay and suspected of having bilateral Blount’s disease. Treatment at 3 years of age consists of hydrocortisone 11.9 mg/m^2^/day and fludrocortisone 0.1 mg/day. ACTH and blood electrolytes are within normal range (Table 2).

### 4.2. Genetic Analysis

Written informed consent was obtained from the parents and genetic studies were performed with ethical approval of the Clinical Research Ethics Committee of the Hospital University Vall d’ Hebron. Genomic DNA was extracted from peripheral blood by standard procedures using the Gentra Pure Gene Blood kit (Qiagen, Madrid, Spain).

A custom panel of genes involved with the biosynthesis of glucocorticoids were analyzed using Gene Read Custom Panel V.2 (Qiagen) technology, which included the exonic regions and ±20 bp intronic boundaries of *AR*, *CYP11A1*, *CYP17A1*, *HSD17B3*, *LHCGR*, *MAMLD1*, *MC2R*, *MCM4*, *NNT*, *NR0B1*, *NR5A1*, *SRD5A2*, *SRY*, *STAR*, *TXNRD2* and *WT1*. DNA libraries were prepared with the NEB Next Ultra DNA Library Prep Kit for Illumina (New England Biolabs, Barcelona, Spain) followed by sequencing in MiSeq (Illumina Inc., San Diego, CA, USA).

Data was analyzed with the following platforms MiSeq control software (MCS), MiSeq reporter (MSR) (both Illumina Inc., San Diego, CA, USA) and Gene Read Targeted Enrichment Exon Panel Data Analysis (Qiagen) software and an in-house variant calling algorithm based in ANNOVAR [41]. Reported *STAR* variants were verified with Sanger sequencing and were classified according to ACMG-AMP guidelines [26]. The variant nomenclature used follows the Human Genome Variation Society (HGVS) guidelines using human STAR NG_011827.1, NM_000349 as reference sequence.

### 4.3. Bioinformatic Studies for Structural and Functional Characterization of Novel Variants Identified in StAR

We prepared the structural model of mutant proteins based on the 3D structure of StAR determined by X-ray crystallography (PDB id: 3POL) [42]. Model building and visualization was done with YASARA [43] and PyMOL [44]. Cholesterol molecule was docked into the StAR protein using Autodoc-VINA [45] and refined by MD simulation using AMBER14 forcefield [46,47] using methods described previously [30]. We predicted the physicochemical stability of the StAR protein for every amino acid replacement using SDM software [48], UMD-predictor [49], SIFT [50], PROVEAN [51], Mutation taster [52], PolyPhen [53], Mutation Assesor [54] and CADD [55]. Steered MD simulation to study release of bound cholesterol by StAR was performed with AMBER14 forcefield using a positive force of 2000 pm/ps2 at 298K. Wt and p.L260R variants of StAR proteins were evaluated for binding of cholesterol.

### 4.4. Functional Studies of Identified Novel StAR Variants In Vitro

Materials: All chemicals were purchased from Sigma-Aldrich (Buchs, Switzerland) unless otherwise stated. The protein estimation was done with the Pierce TM660 protein assay kit (LuBio Science, Lucerne, Switzerland). We used the Immobilon-FL 0.45 µm pore size PVDF membranes (Merck, Schaffhausen, Switzerland) for Western blot. We used IRDye 680RD IgG (H+L) goat anti-rabbit and donkey anti-mouse secondary antibodies (Li-cor Biosciences, Bad Homburg, Germany) to detect primary antibodies in Western blots. We used MG132 (C2211) as a proteasome inhibitor. Non-steroidogenic COS-I cells (CRL-1650TM; http://www.atcc.org) were cultivated in DMEM (Invitrogen, Basel, Switzerland) supplemented with 10% fetal bovine serum, 1% penicillin/streptomycin (100 u/mL, 100 µg/mL) at 37 °C. Vectors expressing cDNAs of wt StAR and a fusion protein CYP11A1-ADXR-ADX (F2 plasmid) were a gift of Walter L. Miller, UCSF, San Francisco [4,56]. The mutant STAR vector was built by PCR-based site-directed mutagenesis.

COS-1 cells were transiently transfected (lipofectamine 2000, Invitrogen, Basel, Switzerland) with wt StAR/variants vectors and F2, and tested for their ability to produce pregnenolone from endogenous cholesterol. Wherever applicable, we treated transfected COS-1 cells with proteasome inhibitor MG132 for 16 h. Forty-eight hours post transfection pregnenolone concentration was measured in the cell supernatants by LC-MS/MS. Briefly, steroids were extracted from 500 µL supernatant using solid phase extraction and analyzed using an Acquity UPLC system coupled to a Q Exactive Plus Orbitrap instrument (both Thermo Fisher, Reinach, Switzerland). For Western blot analysis, cells were collected 48h after transfection and lysed in lysis buffer (EDTA 1 mM, Tris 20 mM, NaCl 150 mM, 1% Triton X-100); Western blot was performed as previously described [30]. For visualization we used Licor Odyssey 9260 fluorescence imager (Li-cor Biosciences, Bad Homburg, Germany).

### 4.5. Statistical Analysis

We present results of three independent experiments as mean ± SEM. Multiple comparison ANOVA test was used for statistical analysis with p value of <0.05 considered statistically significant. Analysis and graphs were made with Graphpad Prism 7 (Graphpad software, La Jolla, CA, USA, www.graphpad.com).

## Figures and Tables

**Table 1:** Pregnenolone production in COS1 cells transfected with fusion protein CYP11A1/ADXR/ADX and StAR wt or variants. Results are given in pregnenolone nmol/L per µg total protein and in percentage of wt StAR.

Pregnenolone Production	wt StAR	StAR-T204R	StAR-L260R	StAR-L260P
Pregnenolone (nmol/L/µg)	5.92	0	0	0.46
% of wt StAR	100	0	0	8

**Table 2:** Biochemical test results of blood investigations. Low or high values are given in bold. Serum steroids were measured by conventional immunoassays. * Timepoint of minipuberty when higher androgens are expected. nd—not detected.

Biochemical Test	Age		Reference Values
	1.5 m *	6 m	
Na (mEq/mL)	133	137	129–143
K (mEq/mL)	**7.4**	5.2	3.7–5.8
ACTH^A^ (pg/mL)	**15,001**	**119.1**	6–48
Renin (µU/mL)	21.15	**160.1**	5.3–99.1
Aldosterone (pg/mL)	34	-	5–90
DHEAS (µg/mL)	nd	-	<1.12 */<0.49
17OH-progesterone (ng/mL)	**0.03**	**<0.03**	0.13–1.06
Androstenedione (ng/mL)	**0.02**	-	0.1–0.37
Testosterone (ng/mL)	**0.04**	<0.03	0.14–3.63 */0.03–0.12
Cortisol (ng/mL)	-	1.1	1–12
Cortisone (ng/mL)	-	1	6.3–56.7
Progesterone (ng/mL)	-	<0.03	0.1–0.26
11-deoxycortisone (ng/mL)	-	<0.03	0.07–0.4
Corticosterone (ng/mL)	-	<0.03	0.8–15
11-deoxycortisol (ng/mL)	-	<0.03	<10

## Abbreviations

StAR	Steroidogenic acute regulatory protein
CAH	Congenital adrenal hyperplasia
POR	P450 oxidoreductase
AI	Adrenal insufficiency
IMM	Inner mitochondrial membrane
OMM	Outer mitochondrial membrane
FGD	Familial glucocorticoid deficiency
ACTH	Adrenocorticotropic hormone
MC2R	Melanocortin receptor 2
MC1R	Melanocortin receptor 1
START	StAR-related lipid transfer
NGS	Next generation sequencing
SNARE	Soluble NSF attachment receptor protein
BMI	Body mass index
FSH	Follicle-stimulating hormone
LH	Luteinizing hormone
ACMG-AMP	American College of Medical Genetics and Genomics and the Association for Molecular Pathology
